# Identification of necroptosis‐related genes in ankylosing spondylitis by bioinformatics and experimental validation

**DOI:** 10.1111/jcmm.18557

**Published:** 2024-07-19

**Authors:** Pengfei Wen, Yan Zhao, Mingyi Yang, Peng Yang, Kai Nan, Lin Liu, Peng Xu

**Affiliations:** ^1^ Department of Joint Surgery, Honghui Hospital Xi'an Jiaotong University Shaanxi China; ^2^ Department of Laboratory, Honghui Hospital Xi'an Jiaotong University Shaanxi China

**Keywords:** ankylosing spondylitis, bioinformatics analysis, ceRNA network, immune cell infiltration, necroptosis

## Abstract

The pathogenesis of ankylosing spondylitis (AS) remains unclear, and while recent studies have implicated necroptosis in various autoimmune diseases, an investigation of its relationship with AS has not been reported. In this study, we utilized the Gene Expression Omnibus database to compare gene expressions between AS patients and healthy controls, identifying 18 differentially expressed necroptosis‐related genes (DENRGs), with 8 upregulated and 10 downregulated. Through the application of three machine learning algorithms—least absolute shrinkage and selection operation, support vector machine‐recursive feature elimination and random forest—two hub genes, FASLG and TARDBP, were pinpointed. These genes demonstrated high specificity and sensitivity for AS diagnosis, as evidenced by receiver operating characteristic curve analysis. These findings were further supported by external datasets and cellular experiments, which confirmed the downregulation of FASLG and upregulation of TARDBP in AS patients. Immune cell infiltration analysis suggested that CD4^+^ T cells, CD8^+^ T cells, NK cells and neutrophils may be associated with the development of AS. Notably, in the group with high FASLG expression, there was a significant infiltration of CD8^+^ T cells, memory‐activated CD4^+^ T cells and resting NK cells, with relatively less infiltration of memory‐resting CD4^+^ T cells and neutrophils. Conversely, in the group with high TARDBP expression, there was enhanced infiltration of naïve CD4^+^ T cells and M0 macrophages, with a reduced presence of memory‐resting CD4^+^ T cells. In summary, FASLG and TARDBP may contribute to AS pathogenesis by regulating the immune microenvironment and immune‐related signalling pathways. These findings offer new insights into the molecular mechanisms of AS and suggest potential new targets for therapeutic strategies.

## 1 INTRODUCTION

Ankylosing spondylitis (AS) is an intractable autoimmune disease characterized by chronic inflammation and aberrant bone growth, affecting approximately 0.1%–0.3% of individuals globally.^1^, ^2^ This condition is particularly prevalent among young men, with a male‐to‐female ratio ranging from 2.3:1 to 3.8:1.^2^, ^3^ Clinical manifestations of AS include inflammation in the sacroiliac joints and spinal regions, leading to back pain, spinal stiffness and deformity.^4^, ^5^, ^6^ While AS predominantly affects the axial skeleton, it can also involve peripheral joints such as the hips.^4^, ^5^ Extra‐skeletal manifestations include acute uveitis, psoriasis and intestinal inflammation.^7^, ^8^ The complexity and highly teratogenic nature of AS present substantial challenges to the medical community,^9^ necessitating a deeper comprehension of its aetiology to devise effective preventative measures and therapeutic strategies.

Necroptosis, a novel kind of programmed cell death, has been shown to play a pivotal role in a variety of immunological diseases, including inflammatory conditions,^10^, ^11^, ^12^ rheumatoid arthritis (RA),^13^ and certain cancers.^14^ Characterized by organelle swelling, disruption of cell membrane integrity and the release of intracellular contents, necroptosis is activated by receptor‐interacting protein kinases 1/3 (RIPK1/3) and mixed lineage kinase domain‐like protein (MLKL), with caspase‐8 being a crucial factor in inhibiting this process.^15^, ^16^, ^17^, ^18^ In RA, necroptosis has been linked to inflammation and joint damage.^19^ Specifically, neutrophils and macrophages within the joints of RA patients can trigger necroptosis under the influence of specific cytokines, leading to exacerbated inflammation and joint injury.^20^ Similarly, in the pathophysiological process of inflammatory bowel disease (IBD), abnormal deaths of intestinal epithelial cells, including necroptosis, may lead to the impairment of intestinal barrier function and the persistence of chronic inflammation.^21^ Studies have confirmed that inhibiting the necroptosis regulatory factors RIPK1/3 can ameliorate intestinal inflammatory symptoms.^22^ It is noteworthy that necroptosis not only triggers inflammatory responses but is also regulated by cytokines within the inflammatory process itself.^23^ Given the close relationship between AS pathogenesis and immune system function state, it is plausible that necroptosis may play a similarly critical role in the pathology of AS.^24^, ^25^, ^26^ This supposition provides a compelling rationale for investigating the relationship between necroptosis and AS.

This study initially screened for necroptosis‐related genes (NRGs) that exhibit differential expression between AS and normal control samples. Utilizing three distinct machine learning algorithms, key biomarkers associated with necroptosis were identified. Subsequently, a systematic analysis of immune cell infiltration in AS was performed and the relationship between immune cells and these hub genes was investigated. Finally, a complex regulatory network involving miRNAs and lncRNAs centered around the identified hub genes was constructed.

Through comprehensive bioinformatics analysis, we aimed to unravel the complex relationship between necroptosis genes and AS, thereby shedding new light on the molecular pathogenesis of the disease. Additionally, our research endeavours to discover novel diagnostic biomarkers and identify targets for immunomodulatory therapies, laying the groundwork for future interventions to alleviate the burden of this disabling condition.

## 2 MATERIALS AND METHODS

### 2.1 Data collection

**FIGURE 1:**
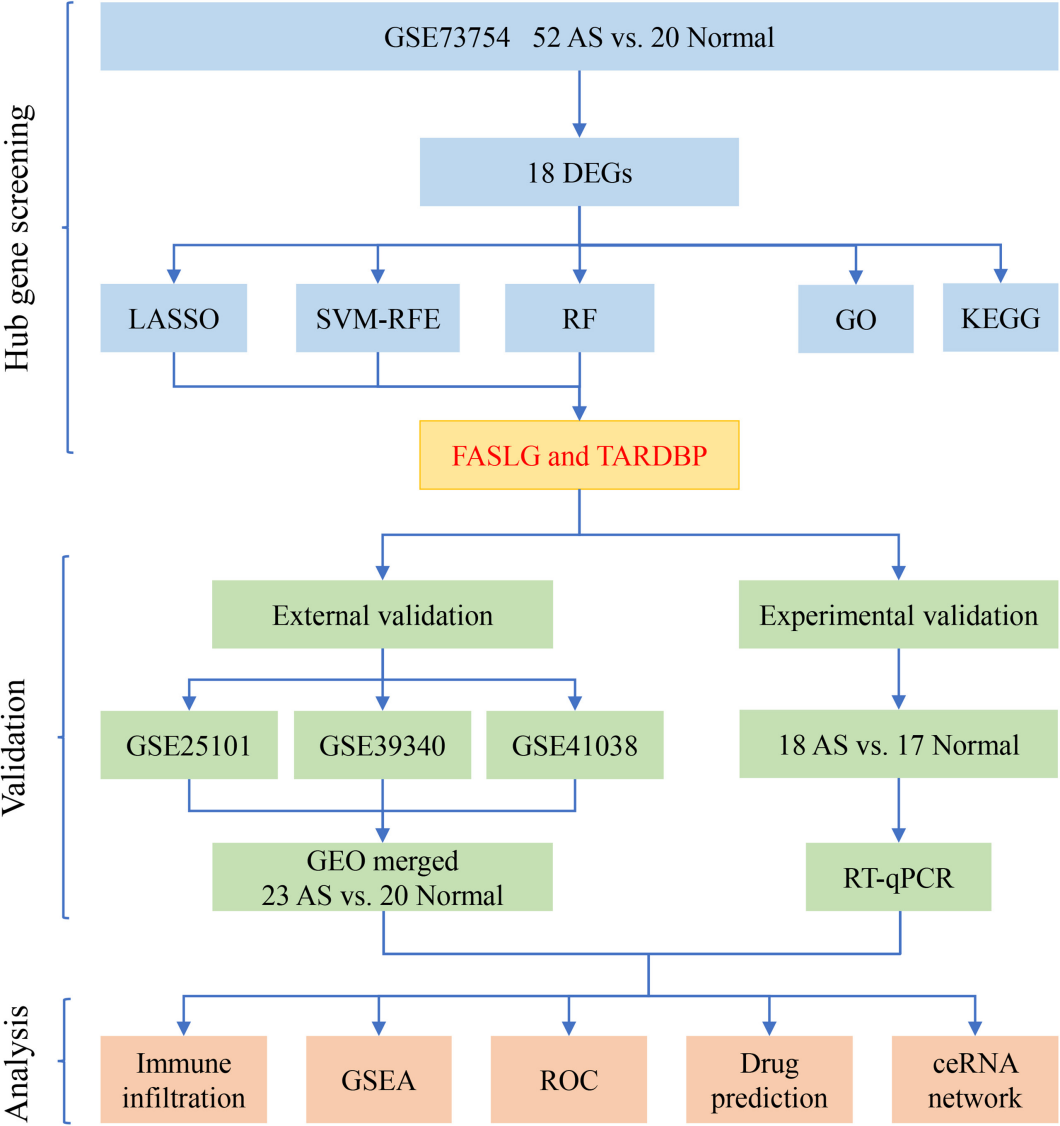
Flowchart of the present study.

Four AS‐related datasets (GSE73754, GSE25101, GSE39340 and GSE41038) were obtained for this investigation from the GEO database (http://www.ncbi.nlms.nih.gov/geo). We used the GSE73754 dataset as the training group, which was constructed based on the GEO platform GPL10558 and comprised 52 samples of AS patients and 20 samples of normal controls.^27^ The datasets GSE25101, GSE39340 and GSE41038 served as validation sets. The GSE25101 dataset included 16 AS patient samples and 16 normal control samples and was constructed based on GPL6947.^28^ The GSE39340 dataset included 5 AS patients and was constructed based on GPL10558.^29^ The GSE41038 dataset was constructed based on the GPL6883 and included samples from two AS patients and four normal controls.^30^ Figure 1 provides a flowchart illustrating the study's methodology.

### 2.2 Screening of differentially expressed NRGs

We extracted expression data for 67 NRGs from both normal control samples and AS samples in the GSE73754 dataset (Table S1). Subsequently, a Student's *t*‐test was performed to obtain the differentially expressed NRGs (DENRGs) in this dataset (R package “limma”). A heatmap of these DENRGs was created using the R package “pheatmap”. Genes with *p* < 0.05 were considered to be significantly differentially expressed.

### 2.3 Functional annotation of DENRGs

We carried out enrichment analyses using the Kyoto Encyclopedia of Genes and Genomes (KEGG) and Gene Ontology (GO) to learn more about the biological roles of NRGs in AS. The R packages “ggplot2”, “clusterProfiler”, “org.Hs.eg.db” and “enrichplot” were used for the enrichment analysis of DENRGs. GO and KEGG terms with a *p*‐value <0.05 were considered significantly enriched.

### 2.4 Screening of necroptosis‐related hub biomarkers

Least absolute shrinkage and selection operation (LASSO) regression, a classical machine learning algorithm, performs dimensionality reduction via the use of a penalty parameter (*λ*). In this work, 10‐fold cross‐validation was carried out to determine the optimal penalty parameter.^31^ We eliminated genes that would overfit the model using the “glmnet” package. In addition, a Support Vector Machine‐Recursive Feature Elimination (SVM‐RFE) model was built using the “svm” package and selected by the average false alarm rate of 10‐fold cross‐validation.^32^ The SVM module was built using the “e1071” package to further screen for NRGs in AS. The random forest (RF) is an algorithm based on the construction of binary trees using recursive partitioning.^33^ We used the “randomForest” package to construct the RF classification model and ranked the NRGs according to the Gini index to filter the characteristic expressed NRGs. The candidate genes selected by the LASSO, SVM‐RFE and RF algorithms were intersected to ultimately produce hub genes.

### 2.5 Diagnostic value assessment and validation of hub genes

To evaluate the diagnostic ability of the hub genes in differentiating AS patients from normal controls, receiver operating characteristic (ROC) curves were plotted and the corresponding areas under ROC curves (AUC) were calculated using the “pROC” R package. Furthermore, the “sva” R package was used to remove batches from validation datasets and merge them for validation of the hub genes. Finally, further collection of blood samples from AS patients was conducted to biologically validate the expression of these genes using RT‐qPCR.

### 2.6 RT‐qPCR validation

Blood samples were collected from 18 AS patients who visited the Department of Joint Surgery at Xi'an Honghui Hospital between January and June 2023. The diagnosis of AS was based on the New York criteria.^34^ Seventeen healthy control samples were included in the comparison analysis. All AS patients and controls participating in the study provided written informed consent. This study was approved by the Ethics Committee of the Xi'an Honghui Hospital. The patients' information is shown in Table 1. The mRNA expression levels of hub genes were analysed by RT‐qPCR. Total RNA was extracted from peripheral venous blood samples using Trizol reagent (Beyotime, Shanghai, China). Reverse transcription was performed using EasyScript One‐Step gDNA Removal and cDNA Synthesis SuperMix (TransGen Biotechnology, Beijing, China). RT‐qPCR amplification was conducted with the Robust SYBR Green qPCR ProMix (EnzyValley, Guangzhou, China) on a StepOnePlus polymerase chain reaction detection system (BIOER, Hangzhou, China). Gene expression levels were calculated using the $2^{-\Delta \Delta C_{t}}$ method. GAPDH was used as an internal reference. The gene‐specific primers used for amplification were as follows: Homo sapiens *FASLG*‐fw: GTAGCTCCTCAACTCACCTAATG. FASLG‐Rv: CCTCAGGTCACAACCATGTATC. Homo sapiens *TARDBP*‐fw: ACGGTTACAGCCCAGTTTC. TARDBP‐rv: GTCACACCATCGTCCATCTATC.

**TABLE 1:** Patient characteristics.

	AS	Control	*p*‐Value
Patients	18	17	
Sex, *n*, female/male	14/4	14/3	1.000
Age, year	32 (21–46)	36 (24–52)	0.171
HLA‐B27, positive, *n*	18/18	0/17	<0.001
ESR, mm/h	34.7 ± 8.6	10.3 ± 5.4	<0.001
CRP, mg/L	22.6 ± 10.1	3.6 ± 1.7	<0.001

Abbreviations: CRP, C‐reactive protein; ESR, erythrocyte sedimentation rate; HLA‐B27, human leucocyte antigen B27.

### 2.7 Gene set enrichment analysis

Gene set enrichment analysis (GSEA) was used to elucidate the biological importance of hub genes from a functional perspective.^35^ This analysis was carried out using the “clusterProfiler” package. The reference gene set was “c2.cp.kegg.v11.0.symbols”.^36^ With FDR <0.05 considered as significantly enriched, 1000 gene set interchanges were conducted to generate the normalized enrichment score for each analysis.

### 2.8 Immune infiltration analysis

CIBERSORT, a systems biology tool that utilizes linear support vector regression (LSVR) machine learning techniques for deconvolution analysis of samples, is capable of accurately assessing the relative abundance of 22 immune cell subtypes from heterogeneous samples (http://cibersortx.stanford.edu).^37^ CIBERSORT has been rigorously validated through fluorescence‐activated cell sorting (FACS) and has demonstrated efficient analytical capabilities in immune profiling for a variety of diseases.^14^, ^31^, ^38^ In dataset GSE73754, we estimated the proportions of 22 immune cells in each sample using the CIBERSORT algorithm. The R package “vioplot” was then employed to generate violin plots that illustrate the shifts in immune cell expression between the AS group and the normal control group. In addition, the “ConsensusClusterPlus” package was used to analyse the differences in the immune microenvironment in the hub gene groups with high and low expression. Graphs of the resulting correlations were shown using the “ggplot2” package.

### 2.9 Drug prediction and competing endogenous RNA network

Drugs targeting hub genes were predicted using the drug–gene interaction database (DGIdb, https://dgidb.org). The interactions between mRNA and miRNA for hub genes were predicted using the starBase database (http://starbase.sysu.edu.cn).^39^ Simultaneously, the mRNA sequences of the hub genes were collected from the National Center for Biotechnology Information (NCBI, https://www.ncbi.nlm.nih.gov) and human miRNA sequences were obtained from miRbase (https://www.mirbase.org). Then, mRNA‐miRNA nucleic acid binding was predicted by miRanda software with a default binding score threshold of 140. Finally, the predicted miRNAs were searched in starBase and screened for miRNA‐lncRNAs, so that a comprehensive ceRNA network of mRNA‐miRNA‐lncRNAs was obtained.

### 2.10 Statistical analysis

The R software version 4.1.0 was used to complete statistical tests. For comparing differences between groups, either the Wilcoxon test or the Student's *t*‐test was employed, depending on the data distribution. Pearson's or Spearman's tests were used to analyse correlations between variables. Statistical differences in patient age, erythrocyte sedimentation rate, C‐reactive protein and RT‐qPCR data were analysed using the *t*‐test in GraphPad Prism 7 (San Diego, CA). The chi‐square test was used for count data. Statistics were defined as significant at *p* < 0.05.

## 3 RESULTS

### 3.1 Identification of DENRGs in the GSE73754

**FIGURE 2:**
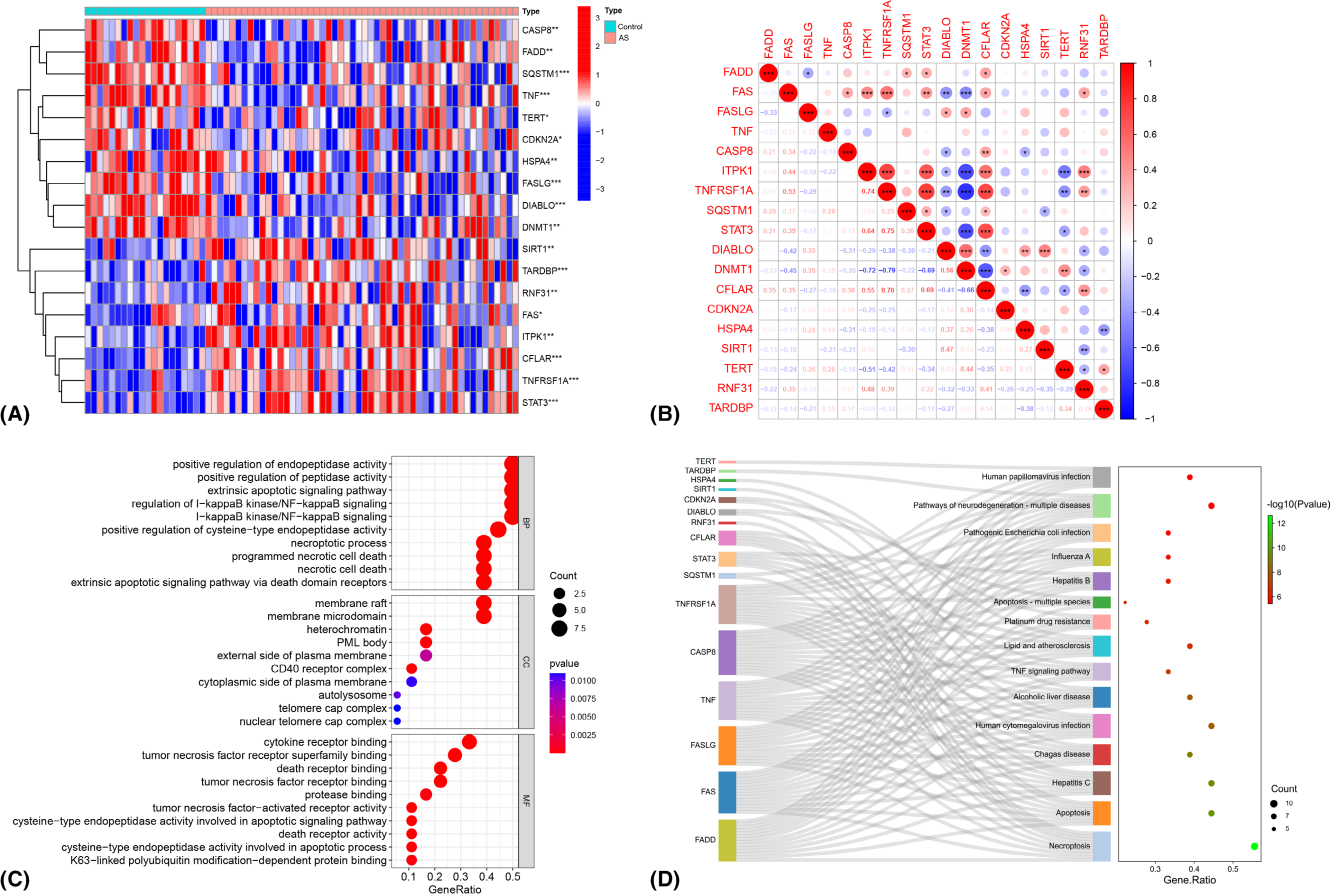
Correlation and enrichment analysis of differentially expressed necroptosis‐related genes (DENRGs) in ankylosing spondylitis (AS). (A) Heat map showing the expression pattern of DENRGs in AS and normal control samples. (B) Correlation analysis of 18 DENRGs. (C) Gene ontology enrichment analysis. (D) Kyoto Encyclopedia of Genes and Genomes enrichment analyses. *p < 0.05, **p < 0.01, ***p < 0.001.

In the GSE73754 dataset, a total of 18 NRGs, including 8 up‐regulated and 10 down‐regulated genes, were discovered to be expressed at significantly different levels between AS cases and healthy control samples (Table 2). The heat map showed the expression pattern of these DENRGs in the samples (Figure 2A). Furthermore, a significant correlation among these genes was revealed by correlation analysis (Figure 2B).

**TABLE 2:** Eighteen differentially expressed necroptosis‐related genes between ankylosing spondylitis and normal control samples.

Gene	ControlMean	ASMean	*p*‐Value	Type
FADD	8.48849145	8.32508125	0.002827173	Down
FAS	7.3807029	7.454304269	0.038627536	Up
FASLG	7.10455085	6.935205365	0.00019326	Down
TNF	7.6569403	7.501242308	0.000150327	Down
CASP8	7.20110515	7.110019423	0.004581679	Down
ITPK1	8.0287278	8.26104725	0.001154257	Up
TNFRSF1A	9.6672127	9.908977846	0.000612564	Up
SQSTM1	11.31845605	11.06418321	1.66E‐05	Down
STAT3	8.37227225	8.586515558	0.000473867	Up
DIABLO	7.8547339	7.745011673	1.97E‐05	Down
DNMT1	10.0939497	9.879554019	0.002112195	Down
CFLAR	10.7896484	11.16252723	7.09E‐05	Up
CDKN2A	6.64458065	6.621631712	0.034673361	Down
HSPA4	7.71541465	7.646614385	0.003466266	Down
SIRT1	8.0870149	8.181366327	0.008441446	Up
TERT	6.59173775	6.570149596	0.043607524	Down
RNF31	8.05965205	8.163879135	0.00925539	Up
TARDBP	7.2245656	7.353735731	0.000364927	Up

### 3.2 Enrichment analysis of DENRGs

Biological processes and pathways associated with DENRGs in AS were elucidated using GO and KEGG enrichment analysis. The GO analysis revealed that the biological processes (BP) significantly enriched for DENRGs included positive regulation of endopeptidase/peptidase activity, extrinsic apoptotic signalling pathway, I‐kappaB kinase/NF‐kappaB signalling, necroptotic process and programmed necrotic cell death. In terms of cellular components (CC), the membrane raft and membrane microdomain were notably enriched. Moreover, the molecular functions (MF) that were significantly enriched comprised cytokine receptor binding, tumour necrosis factor receptor superfamily binding and death receptor binding (Figure 2C). The KEGG enrichment analysis showed that DENRGs were mainly enriched in pathways related to necroptosis, apoptosis, TNF signalling pathway, neurodegeneration‐multiple diseases, platinum drug resistance, lipid and atherosclerosis, alcoholic liver disease and viral infection‐related pathways (Figure 2D). These results indicated that DENGRs may contribute to the pathogenesis of AS through some of the above‐mentioned signalling pathways.

### 3.3 Identification of diagnostic hub genes for AS

**FIGURE 3:**
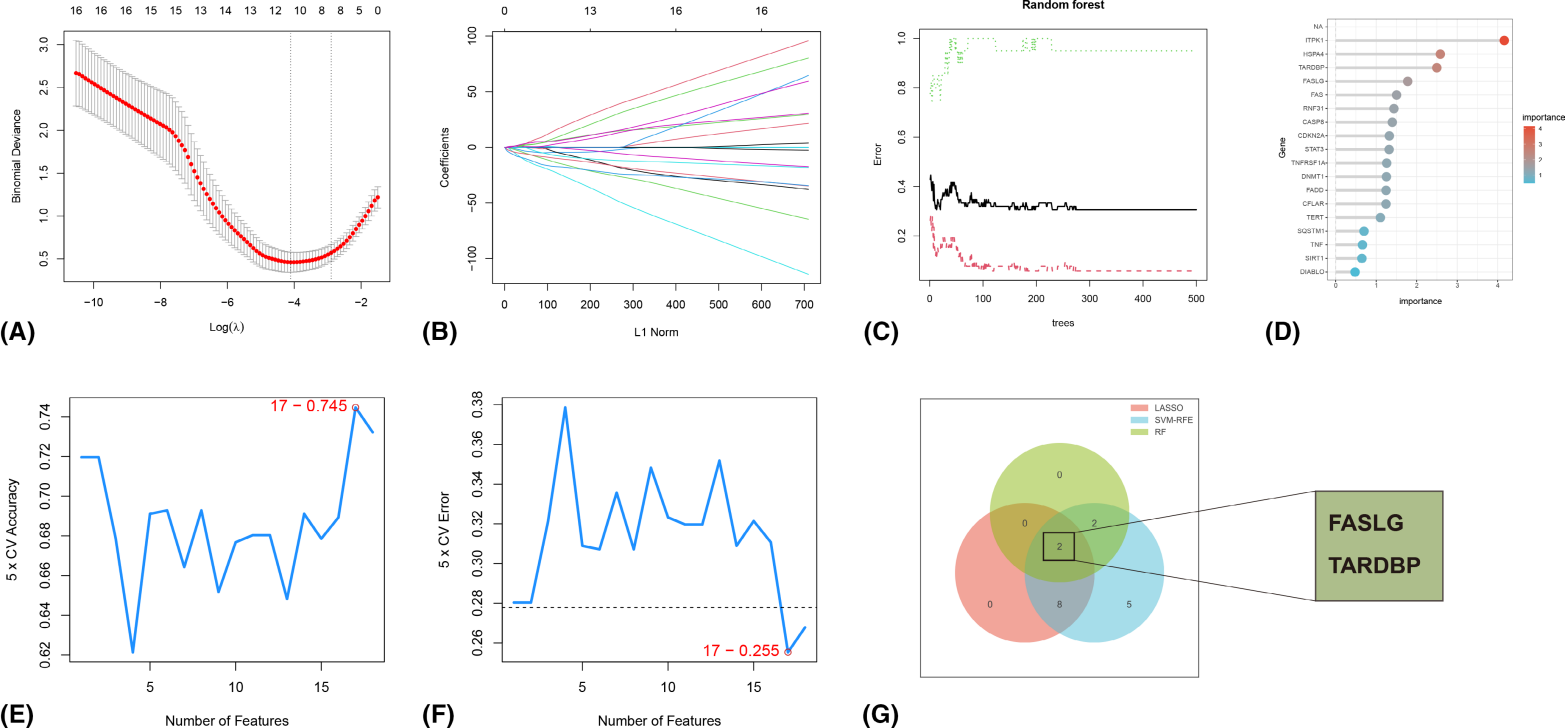
Screening of hub genes. (A, B) Ten candidate genes were obtained by the least absolute shrinkage and selection operation logistic regression algorithm. (C, D) Four candidate genes screened based on gene importance using the random forest algorithm. (E, F) Seventeen candidate genes obtained by the support vector machine‐recursive feature elimination algorithm. (G) Venn diagram of the intersection of candidate genes obtained by three machine learning algorithms.

Due to the differential expression of NRGs between AS patients and healthy individuals, we further obtained hub NRGs with potential diagnostic value for AS. In the GSE73754 dataset, three machine‐learning methods were employed to select meaningful candidate NRGs. Ten of the 18 DENRGs were selected by the LASSO logistic regression algorithm (Figure 3A,B). Meanwhile, based on the gene importance obtained by the RF algorithm, a total of 4 candidate genes were obtained with a screening condition of importance >1.5 (Figure 3C,D). In addition, 17 candidate NRGs were screened by the SVM‐RFE algorithm (Figure 3E,F). Ultimately, by intersecting the candidate genes derived from the aforementioned three algorithms, we pinpointed two hub genes, *FASLG* and *TARDBP*, as shown in Figure 3G. These genes were consistently identified as key by all three methods, suggesting their robust potential as diagnostic markers for AS.

### 3.4 Evaluation and validation of the diagnostic ability of the hub genes

**FIGURE 4:**
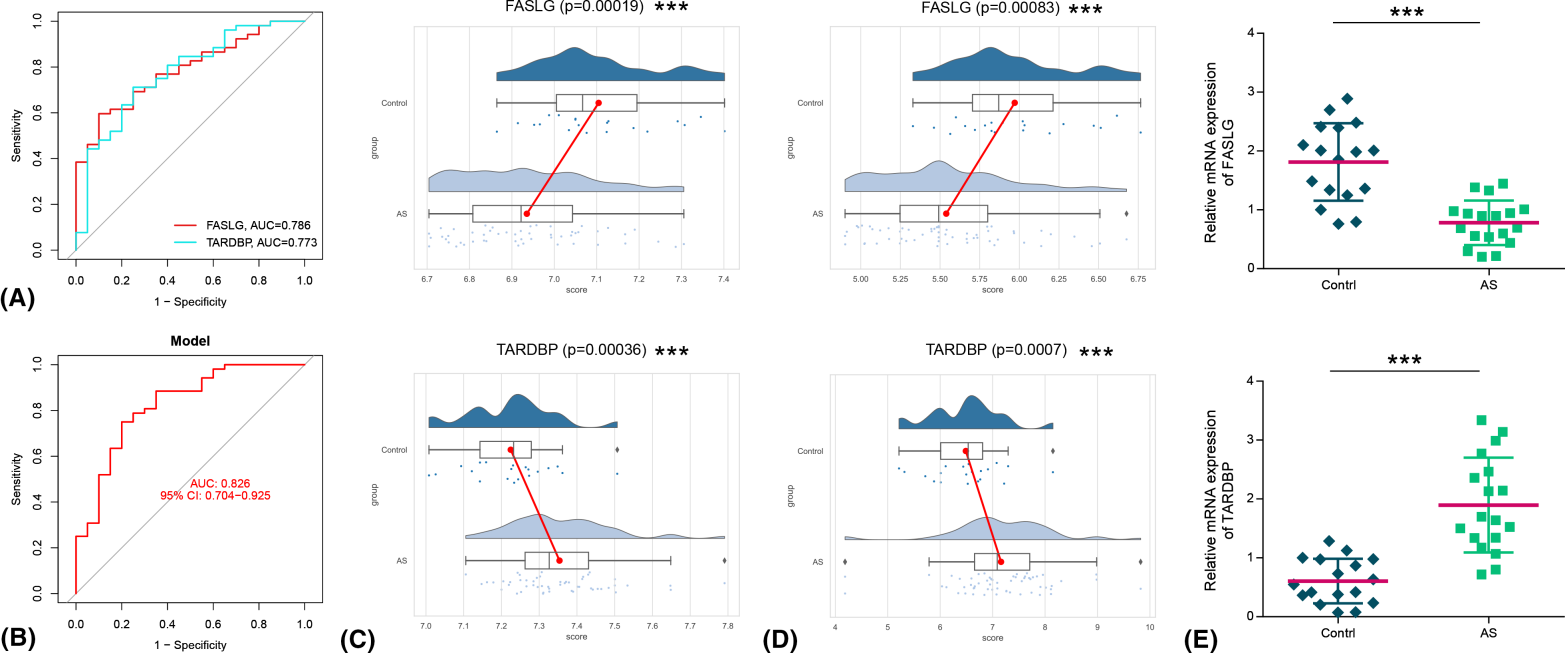
Diagnostic capability assessment and validation of hub genes. (A) Receiver characteristic curves (ROCs) of each hub gene. (B) ROC curve of the logistic regression model based on hub genes. (C) The expression of hub genes in the training set. (D) The expression of hub genes in the validation set. AUC, area under the curve. (E) The mRNA expression of hub genes. ***p < 0.001.

We produced ROC curves for the two hub genes to evaluate their diagnostic capacity for distinguishing AS samples from healthy controls. As depicted in Figure 4A, both hub genes demonstrated high accuracy, with AUCs exceeding 0.75, indicating their potential as reliable biomarkers for distinguishing AS from normal control samples. Subsequently, a logistic regression model incorporating these two hub genes was constructed. The results showed that the model was more accurate and specific in diagnosing AS than a single hub gene alone (Figure 4B). Then, the expression of the hub genes in the validation datasets (GSE25101, GSE39340 and GSE41038) was analysed. The results showed that the hub genes (*FASLG* and *TARDBP*) had similar expression trends in both the training and validation sets. Specifically, *FASLG* expression was downregulated, while *TARDBP* expression was upregulated in AS patients compared to normal controls (Figure 4C,D). Finally, the mRNA expression levels of *FASLG* and *TARDBP* in AS patients and healthy controls were detected by RT‐qPCR. The analysis confirmed that the expression trends of *FASLG* and *TARDBP* were consistent with the bioinformatics analysis (Figure 4E). This validation reinforces the reliability of the hub genes as diagnostic markers for AS.

### 3.5 GSEA analysis of hub genes

**FIGURE 5:**
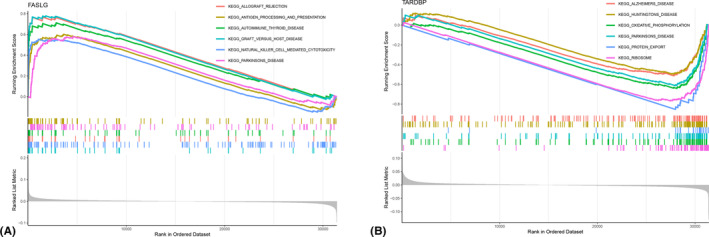
Gene set enrichment analysis enrichment analysis. (A) Result of GSEA analysis of FASLG and (B) TARDBP.

Hub genes were subjected to GSEA analysis to better investigate their potential pathways and functions in AS samples between high and low‐expression groups. Figure 5A,B highlights the pathways significantly enriched for each hub gene. Specifically, *FASLG* was predominantly enriched in KEGG pathways associated with Parkinson's disease, autoimmune thyroid disease, graft‐versus‐host disease, antigen processing and presentation and allograft rejection. *TARDBP* was primarily enriched in Alzheimer's disease, Huntington's disease, oxidative phosphorylation, Parkinson's disease, protein export and ribosome pathways. This GSEA analysis provides a clearer understanding of the biological processes and pathways that may be influenced by the expression levels of *FASLG* and *TARDBP* in AS.

### 3.6 Immune microenvironment and AS

**FIGURE 6:**
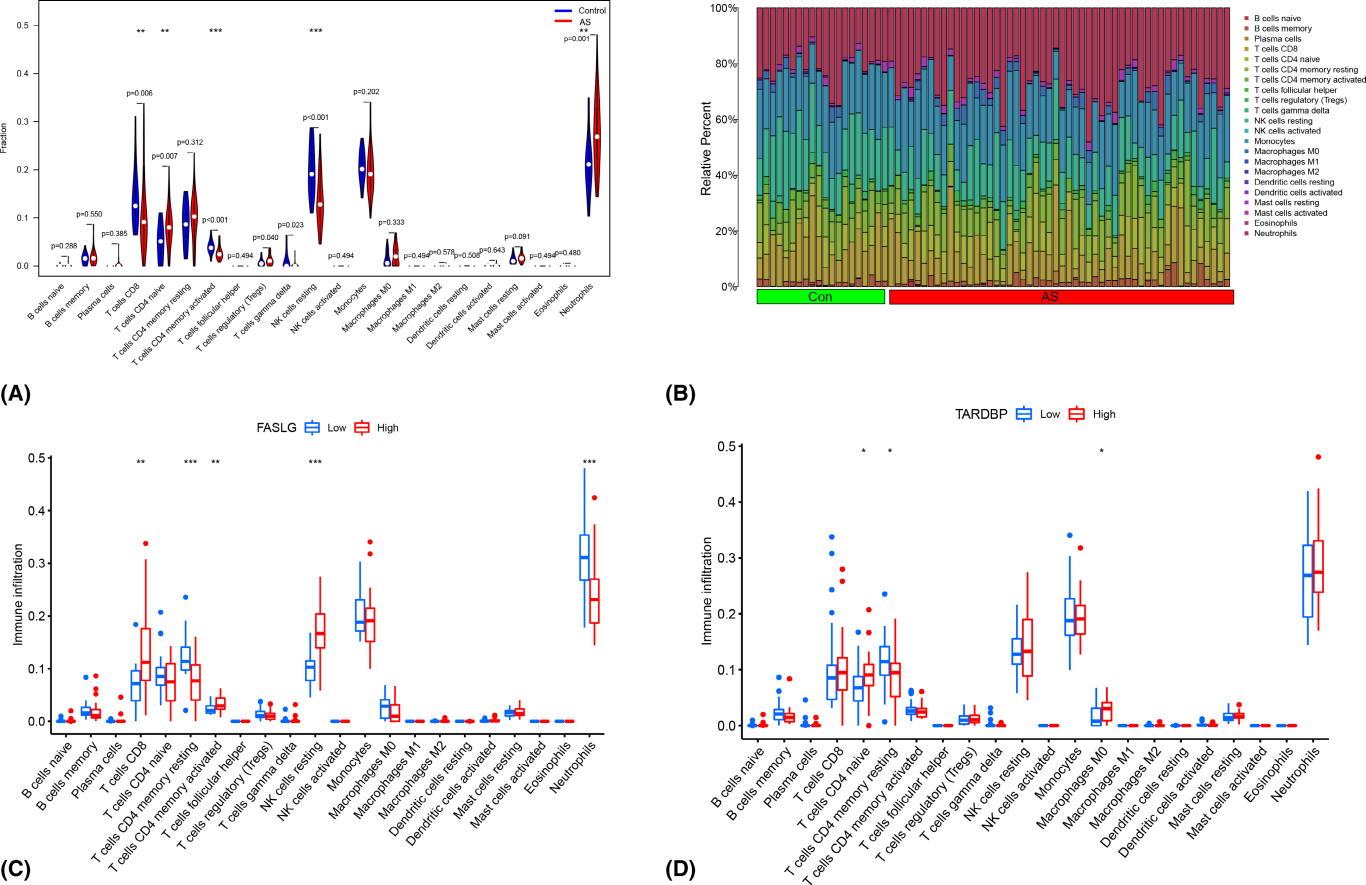
Immune cell infiltration analysis. (A) Differences in immune microenvironment between ankylosing spondylitis samples and normal control samples explored by the CIBERSORT algorithm. (B) Histogram of the proportional distribution of 22 immune cells in all samples. (C) Differences in immune microenvironment in the high and low expression groups of FASLG. (D) Differences in immune microenvironment in the high and low expression groups of TAROBP. *p < 0.05, **p < 0.01, ***p < 0.001.

Increasing evidence has demonstrated the intricate connection between the immune microenvironment and AS. To explore the differences in immune cell infiltration between AS and control samples, the CIBERSORT method was employed. Figure 6A,B illustrates that AS samples have a significantly reduced proportion of CD8^+^ T cells, memory‐activated CD4^+^ T cells and resting NK cells compared to normal control samples. Conversely, AS samples exhibit increased infiltration of naïve CD4^+^ T cells, regulatory T cells and neutrophils. Further examination of the immunological microenvironment in groups with high and low expression of the hub genes revealed distinct patterns. In the group with high *FASLG* expression, there was enhanced infiltration of CD8^+^ T cells, memory‐activated CD4^+^ T cells and resting NK cells, whereas memory‐resting CD4^+^ T cells and neutrophils were comparatively less infiltrated (Figure 6C). In the group with high *TARDBP* expression, naïve CD4^+^ T cells and Macrophages M0 were more infiltrated, while memory‐resting CD4^+^ T cells were less infiltrated (Figure 6D). The above results suggest that the altered immune microenvironment in AS patients may be closely related to the expression level of *FASLG* and *TARDBP*.

### 3.7 Prediction of hub gene‐targeted drugs and construction of ceRNA network

**FIGURE 7:**
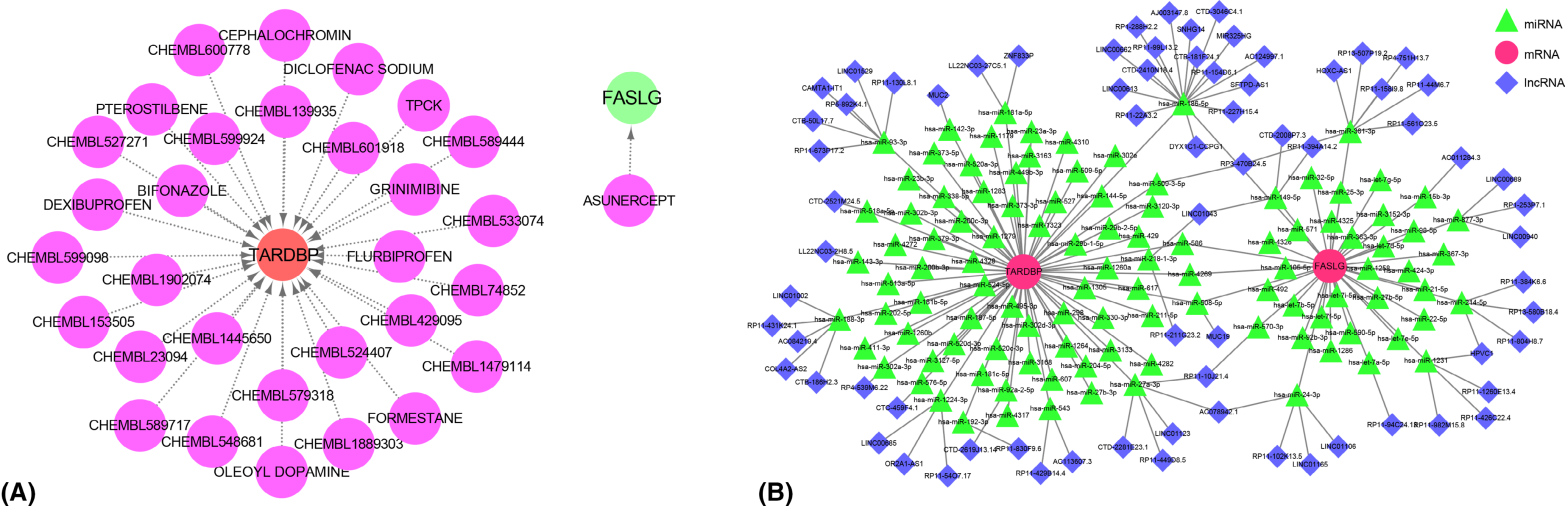
Targeted drug prediction for hub genes and the ceRNA network based on hub genes. (A) Targetable drugs for hub genes. The purple circles represent predicted drugs. (B) Competing endogenous RNA interaction network, including 182 nodes (2 hub genes, 107 miRNAs and 73 lncRNAs) and 183 edges.

The DGIdb database and Cytoscape software were used to find potential medications targeting the hub genes, as depicted in Figure 7A. We retrieved 31 drugs that target these genes, with only one drug affecting FASLG and the remaining 30 targeting TARDBP (Figure 7A). In addition, a ceRNA network based on the two hub genes was constructed through the starBase and miRanda databases. This network included 182 nodes (2 hub genes, 107 miRNAs and 73 lncRNAs) and 183 edges (Figure 7B). By competitively binding to miRNAs, 23 lncRNAs can regulate *FASLG*, 29 lncRNAs can regulate *TARDBP* and 21 lncRNAs can regulate both hub genes. In detail, lncRNA RP11‐10J21.4 can target miR‐570‐3p and miR‐27a‐3p; AC078942.1 can target miR‐27a‐3p and miR‐24‐3p. Moreover, LINC01043 can compete for miR218‐1‐3p and miR‐149‐5p; RP3‐470B24.5 can compete for miR‐509‐3‐5p and miR‐361‐3p, simultaneously. Notably, within the ceRNA network, 16 lncRNAs can bind to miR‐186‐5p to regulate both hub genes, while MUC19 can competitively bind to miR‐508‐5p for the regulation of both *FASLG* and *TARDBP*. This comprehensive analysis not only elucidates the complex interactions within the ceRNA network but also highlights the potential therapeutic implications of modulating these gene expressions in AS.

## 4 DISCUSSION

Ankylosing spondylitis is an intractable and highly disabling autoimmune disease that affects the spine and joints, posing ongoing challenges for early diagnosis and treatment.^1^ The advancement of bioinformatics in recent years has significantly propelled the exploration of potential diagnostic markers and therapeutic targets for AS. Ni et al. discovered that programmed cell death 10 (*PDCD10*) is upregulated in AS patients, promoting the calcification of synovial cells in vitro and showing a positive correlation with prognostic parameters of AS.^40^ Li et al.^41^ identified the pyroptosis‐related gene Granzyme B (*GZMB*) plays an important role in AS. Jiang et al.^42^ found that the expression of *ANXA3* and *SORT1L* in AS is significantly higher than in the control group, with a positive correlation to neutrophil counts. However, these markers have not yet seen widespread application. This study diverges markedly from previous ones by focusing on the role of necroptosis in AS for the first time. Utilizing advanced bioinformatics methods and machine learning algorithms, we have sifted through extensive data to identify DEGs associated with necroptosis. Further analysis pinpointed two hub genes: *FASLG* and *TARDBP*, which exhibit notable expression differences in AS patients and demonstrate high accuracy in distinguishing AS from normal controls. Crucially, our findings have been corroborated by external datasets and qt‐PCR, which significantly enhances the credibility of our research. Moreover, our results offer new insights and potential biomarkers for the early diagnosis and treatment of AS.

To reveal the role of NRGs in AS, enrichment analysis has identified key terms including necroptosis/apoptotic signalling pathway, programmed necrotic cell death, TNF receptor family, TNF signalling pathway, lipid metabolism, I‐kappaB/NF‐kappaB signalling pathway and viral infection‐related pathways. Necroptosis, a newly identified form of programmed cell death, is activated by RIPK1/3 and MLKL.^15^, ^16^ TNF‐α is recognized as a mediator of systemic immune responses, with its receptor, TNF‐receptor1 (TNFR1), capable of inducing the binding of Fas‐associated death domain (FADD) to the TNFR1‐associated death domain (TRADD) and RIPK1, thereby activating necroptosis through an MLKL/RIPK3‐dependent mechanism.^43^, ^44^ TNF signalling pathways are involved in AS pathogenesis from early inflammation to advanced stages of bone formation. High levels of TNF were found in sacroiliitis during the early stages of AS.^45^ The binding of TNF with TNFR1 can lead to the recruitment of TRADD, RIPK1 and TNFR‐associated factor 2 (TRAF2) to form a complex. This in turn triggers some phosphorylation and ubiquitination events that lead to the activation of nuclear factor κB (NFκB), a central mediator of the pro‐inflammatory effects of TNF^43^. One study found higher expression levels of the IKBα and NFκB in AS patients compared to healthy subjects.^46^ Furthermore, the downregulation of LOC645166 in the T cells of AS patients could enhance NFκB activation, increasing their sensitivity to pro‐inflammatory cytokines.^47^ TNF may also promote osteogenic differentiation and pathological bone formation by activating NFκ in AS.^44^ In addition, TNF‐α inhibitors have proven highly effective in reducing inflammation in AS patients,^48^ with the response to these inhibitors potentially influenced by infection‐related and metabolic pathways.^49^ The above evidence suggests that DENRGs may be crucial in the aetiology of AS via certain signalling pathways.

The *FASLG* (Fas ligand) gene, a member of the TNF family, encodes a transmembrane protein that primarily functions to induce apoptosis by binding to Fas.^50^ The Fas/FasL signalling pathway plays a crucial role in immune system regulation, including cytotoxic T lymphocyte‐induced cell death, T cell activation‐induced cell death and NK cell‐mediated apoptosis.^51^, ^52^ Microarray analysis of joint tissue from arthritic mice showed that the Faslg‐DR5 interaction could drive autoantibody‐induced joint inflammation.^53^ Defects of *FASLG* are closely associated with some autoimmune and inflammatory diseases, such as systemic lupus erythematosus (SLE),^54^ polymyositis,^55^ and autoimmune lymphoproliferative syndrome.^56^ Soluble forms of Fas (sFas) and FasL (sFasL) have been reported to be increased in the serum of SLE patients compared to healthy controls.^54^ Furthermore, serum sFas levels were found to correlate with active renal SLE and disease flares, while the sFasL/sFas ratio was negatively correlated with disease activity and cumulative organ damage.^54^ In RA patients, sFasL was found to be elevated in synovial fluid and correlated with the aggressiveness of the pathology and disease progression.^57^ Notably, Fas activation can stimulate the synthesis of inflammatory cytokines, which participate in the activation and recruitment of innate immune cells, such as neutrophils and macrophages.^58^ In antigen‐presenting cells, Fas can activate caspase‐1 and produce IL‐1β through a caspase‐8‐dependent pathway when combined with TNF.^57^, ^58^ The GSEA analysis in this study also revealed the enrichment of *FASLG* in multiple immune pathways (Figure 4A). Given these results, it is suggested that *FASLG* may be involved in the pathophysiology of AS through the regulation of immune cell apoptosis.

The *TARDBP* gene, which encodes the transactive response DNA‐binding protein of 43 kDa (TDP‐43), is recognized as crucial in the pathogenesis of frontotemporal dementia (FTD), amyotrophic lateral sclerosis (ALS) and motor neuron disease.^59^, ^60^ Bright et al.^59^ provided a detailed review of the relationship between TDP‐43 and the innate immune complement cascade, as well as central innate immune‐inflammatory pathways including NF‐κβ/p65, NLRP3 inflammasome, MAPK/JNK/p38 and cGAS/STING. Importantly, adaptive immune alterations were found to be associated with TDP‐43 in ALS and FTD.^59^ Many population‐based studies have found AS as a risk factor for Parkinson's disease, with a significantly higher prevalence of Alzheimer's among AS patients,^61^, ^62^ which agrees with the GSEA analysis findings in the current study. Moreover, recent studies identified that *TARDBP* would be a novel NRG in the tumour microenvironment.^63^, ^64^ However, studies correlating *TARDBP* with AS are rare, and the role of *TARDBP* in AS requires further experimental studies.

It is well established that a variety of immune cells are involved in the onset, progression and treatment of AS.^65^ In the present study, the infiltration of CD8^+^ T cells, CD4^+^ T cells, regulatory T cells, NK cells and neutrophils were identified to be significantly different between the normal and AS groups, which is in agreement with previous research.^66^ Compared to healthy controls, AS patients exhibited higher numbers of naïve CD4^+^ T cells, regulatory T cells and neutrophils, while showing lower proportions of memory‐activated CD4^+^ T cells, CD8^+^ T cells and resting NK cells. Studies have found that the blood levels of regulatory T cells in AS patients are significantly lower than in healthy controls.^67^ Therapies utilizing TNF‐α inhibitors for AS treatment may work by rebalancing the immune system's CD4^+^ T cell and regulatory cell populations.^68^ Additionally, a large‐scale study of peripheral blood tests revealed that AS patients have considerably higher neutrophil counts than healthy controls.^42^ A recent meta‐analysis further confirmed the upregulation of neutrophils in AS.^69^ IL‐17A, expressed on neutrophil outer traps in AS, could promote the osteogenic differentiation of mesenchymal stem cells.^70^ In further immune infiltration analysis between high and low expression groups of hub genes (*FASLG* and *TARDBP*), we found increased infiltration of CD8^+^ T cells, memory‐activated CD4^+^ T cells and NK cells in the *FASLG* high expression group. In contrast, the *TARDBP* high expression group showed greater infiltration of naïve CD4^+^ T cells and macrophage M0. These findings suggest that the altered immune microenvironment in AS patients may be regulated by the expression levels of these hub NRGs. Thus, assessing immune cell infiltration is essential for understanding the molecular immunological mechanisms behind AS and for developing new immunotherapeutic targets.

MicroRNAs (miRNAs) play an increasingly vital role in various key biological processes, including cell development, proliferation, differentiation, apoptosis and signal transduction.^71^ Chen and Huang have thoroughly discussed the functions of miRNAs, their interactions with target genes, miRNA‐disease associations (MDAs) and several important publicly available miRNA‐related databases.^71^, ^72^ Identifying disease‐associated miRNAs can facilitate the understanding of disease mechanisms at the molecular level and accelerate the design of diagnostic, therapeutic, and preventive molecular tools. For instance, several miRNAs have been identified with diagnostic and therapeutic potential in prostate cancer.^72^ Necroptosis‐related genes have also been identified in the progression of prostate cancer,^73^ suggesting that miRNAs might regulate these genes, thereby affecting treatment strategies. Recently, Huang and colleagues assessed 29 advanced computational models for predicting MDA and proposed an evaluation process to enhance predictive performance.^74^, ^75^ This contributes to the selection of promising miRNA‐disease pairs, thereby reducing experimental time and costs.

In our study, ceRNA network analysis indicated that miR‐186‐5p has a higher centrality; it is competitively bound by 16 lncRNAs and can simultaneously regulate two hub NRGs. Overexpression of miR‐186‐5p can inhibit cell proliferation, vitality and migration while increasing apoptosis.^76^ It plays a significant role in vascular endothelial injury,^74^ myocardial ischemia,^76^ and tumorigenesis.^77^ However, no studies have yet explored the connection between miR‐186‐5p and hub NRGs. Regis and colleagues found that miR‐24‐3p functionally interacts with *FASLG* in NK cells and downregulates its expression, helping to control both intrinsic and extrinsic apoptotic pathways.^78^ Additionally, inhibition of miR‐24‐3p increases the activity of initiator caspase‐8, enhancing NK cell apoptosis.^78^ MicroRNA‐149‐5p inhibition can target the upregulation of *FASLG* to induce apoptosis in the acute myeloid leukemia cell line THP‐1.^79^ Notably, MUC19 can competitively bind to miR‐508‐5p for the regulation of *FASLG* and *TARDBP*. In short, the complex regulation of hub NRGs in AS still has many gaps. Further studies of the ceRNA network would contribute to the understanding of AS pathogenesis at the genetic level and be expected to develop new diagnostic markers and therapeutic targets for AS.

This study offered several advantages. Firstly, it employed three machine learning algorithms—LASSO, SVM‐RFE and RF—to precisely identify hub genes FASLG and TARDBP with high specificity and sensitivity for diagnosing AS, the accuracy of which was confirmed by ROC curve analysis. Secondly, external datasets and cellular experiments further substantiated the abnormal expression of these genes in AS patients. Lastly, immune infiltration analysis uncovered the role of immune cells in the pathogenesis of AS, providing a novel perspective for understanding the disease's mechanisms. This methodological framework is also applicable to other autoimmune diseases, potentially revealing shared or distinct pathological processes. Of course, there are some limitations in this study. First, the lack of functional experimental validation was the main limitation of this study. Although external datasets and RT‐qPCR experiments provide preliminary support for the findings, the molecular mechanisms of hub genes need to be further explored in vivo and in vitro studies. Second, the data sources used in this study were limited and the sample size was relatively small. Third, gene expression levels may be affected by inflammatory status and a variety of other confounding factors. Therefore, research with a larger sample size and more detailed clinical data will help to illustrate the specific roles of hub genes in the pathogenesis of AS.

## 5 CONCLUSION

In summary, this study identified two hub NRGs, *FASLG* and *TARDBP*, in AS by bioinformatics methods. They demonstrated good diagnostic capability for AS and possible involvement in the pathogenesis of AS by regulating the infiltration of immune cells in the immune microenvironment and relevant signalling pathways through their expression. In addition, the construction of a ceRNA network for these hub NRGs has shed light on potential therapeutic targets for AS. To validate the findings of this investigation, additional prospective basic research is required in future studies.

## AUTHOR CONTRIBUTIONS

**Pengfei Wen:** Conceptualization (lead); data curation (lead); funding acquisition (lead); software (equal); visualization (equal); writing – original draft (lead); writing – review and editing (equal). **Yan Zhao:** Data curation (equal); methodology (equal); software (equal); visualization (equal); writing – review and editing (equal). **Mingyi Yang:** Formal analysis (equal); validation (equal). **Peng Yang:** Formal analysis (equal); validation (equal). **Kai Nan:** Formal analysis (equal); validation (equal). **Lin Liu:** Data curation (equal); methodology (equal). **Peng Xu:** Conceptualization (equal); writing – review and editing (equal).

## FUNDING INFORMATION

This work was supported by the Youth Cultivation Project of Xi'an Health Commission (Program No. 2023qn17) and the Key Research and Development Project of Shaanxi Province (No. 2023‐YBSF‐099).

## CONFLICT OF INTEREST STATEMENT

The authors declare that there are no conflicts of interest.

## Supporting information


Table S1.

## Data Availability

Publicly available datasets (GSE73754, GSE25101, GSE39340 and GSE41038) analysed in this study can be obtained from the GEO (http://www.ncbi.nlm.nih.gov/geo) database. The R code utilized in this study can be accessed and downloaded from GitHub (https://github.com/eavae/Necroptosis‐related_genes_in_AS/tree/main).
