# Supplementary material for: Identification of necroptosis‐related genes in ankylosing spondylitis by bioinformatics and experimental validation
Source: J Cell Mol Med. 2024 Jul 19;28(14):e18557. doi: 10.1111/jcmm.18557 (PMC11258886; doi:10.1111/jcmm.18557)
Supplement: Supplementary file 1 — Table S1. [file JCMM-28-e18557-s001.docx]

**Supplementary table 1.** The 67 necroptosis-related genes.

| Genes | Source |
| --- | --- |
| FADD | necroptosis geneset M24779.gmt |
| FAS | necroptosis geneset M24779.gmt |
| FASLG | necroptosis geneset M24779.gmt |
| MLKL | necroptosis geneset M24779.gmt |
| RIPK1 | necroptosis geneset M24779.gmt |
| RIPK3 | necroptosis geneset M24779.gmt |
| TLR3 | necroptosis geneset M24779.gmt |
| TNF | necroptosis geneset M24779.gmt |
| TSC1 | PMID: 31961824; PMCID: PMC7108921. |
| TRIM11 | PMID: 31961824; PMCID: PMC7108921. |
| CASP8 | PMID: 31748744. |
| ZBP1 | PMID: 31076724; PMCID: PMC7109092. |
| MAPK8 | PMID: 31706322; PMCID: PMC6842489. |
| IPMK | PMID: 29883610; PMCID: PMC5994928. |
| ITPK1 | PMID: 29883610; PMCID: PMC5994928. |
| SIRT3 | PMID: 31894331. |
| MYC | PMID: 32753382; PMCID: PMC7443878. |
| TNFRSF1A | PMID: 27049944; PMCID: PMC4833566. |
| TNFSF10 | PMID: 27049944; PMCID: PMC4833566. |
| TNFRSF1B | PMID: 26993379. |
| TRAF2 | PMID: 26993379. |
| PANX1 | PMID: 31410978; PMCID: PMC6776911. |
| OTULIN | PMID: 29950720. |
| CYLD | PMID: 28362430; PMCID: PMC5520167. |
| USP22 | PMID: 33369872; PMCID: PMC7857539. |
| MAP3K7 | PMID: 27219062; PMCID: PMC4886731. |
| SQSTM1 | PMID: 27219062; PMCID: PMC4886731. |
| STAT3 | PMID: 32100392; PMCID: PMC7171322. |
| DIABLO | PMID: 27194728. |
| DNMT1 | PMID: 32554751; PMCID: PMC7416440. |
| CFLAR | PMID: 30518925; PMCID: PMC6281604. |
| BRAF | PMID: 30157175; PMCID: PMC6114281. |
| AXL | PMID: 30157175; PMCID: PMC6114281. |
| ID1 | PMID: 32004572. |
| CDKN2A | PMID: 28811972; PMCID: PMC5543818. |
| HSPA4 | PMID: 32156734; PMCID: PMC7104336. |
| BCL2 | PMID: 33239070; PMCID: PMC7687715. |
| STUB1 | PMID: 29686306; PMCID: PMC5913227. |
| FLT3 | PMID: 30828789. |
| HAT1 | PMID: 29535128. |
| SIRT2 | PMID: 29535128. |
| SIRT1 | PMID: 29535128. |
| PLK1 | PMID: 22890325; PMCID: PMC3499666. |
| MPG | PMID: 30755477; PMCID: PMC7150588. |
| BACH2 | PMID: 31918262. |
| GATA3 | PMID: 31918262. |
| MYCN | PMID: 26633716; PMCID: PMC4720889. |
| ALK | PMID: 26633716; PMCID: PMC4720889. |
| ATRX | PMID: 26633716; PMCID: PMC4720889. |
| TERT | PMID: 26633716; PMCID: PMC4720889. |
| SLC39A7 | PMID: 30237509; PMCID: PMC6748104. |
| SPATA2 | PMID: 27545878; PMCID: PMC5009064. |
| RNF31 | PMID: 27545878; PMCID: PMC5009064. |
| IDH1 | PMID: 28564603. |
| IDH2 | PMID: 28564603. |
| KLF9 | PMID: 30348136; PMCID: PMC6198521. |
| HDAC9 | PMID: 30348136; PMCID: PMC6198521. |
| HSP90AA1 | PMID: 23147571. |
| LEF1 | PMID: 22157808. |
| BNIP3 | PMID: 20963496. |
| CD40 | PMID: 26313915; PMCID: PMC4558516. |
| BCL2L11 | PMID: 24561519. |
| EGFR | PMID: 25688715. |
| DDX58 | PMID: 33852834; PMCID: PMC8109599. |
| TARDBP | PMID: 33852834; PMCID: PMC8109599. |
| APP | PMID: 34105277; PMCID: PMC8188212. |
| TNFRSF21 | PMID: 34105277; PMCID: PMC8188212. |
